# Structural parameter determination and pruning pattern analysis of pear tree shoots for dormant pruning

**DOI:** 10.1016/j.plaphe.2025.100136

**Published:** 2025-11-13

**Authors:** Jiaqi Li, Hao Sun, Gengchen Wu, Hu Xu, Shutian Tao, Wei Guo, Kaijie Qi, Hao Yin, Shaoling Zhang, Seishi Ninomiya, Yue Mu

**Affiliations:** a Academy for Advanced Interdisciplinary Studies, Collaborative Innovation Center for Modern Crop Production Co-Sponsored by Province and Ministry, State Key Laboratory of Crop Genetics & Germplasm Enhancement and Utilization, Nanjing Agricultural University, Nanjing, 210095, China; b Sanya Institute, College of Horticulture, Nanjing Agricultural University, Nanjing, Jiangsu, 210095, China; c Graduate School of Agricultural and Life Sciences, The University of Tokyo, 1-1-1 Midori-cho, Tokyo, 188-0002, Japan; d College of Horticulture, Xinjiang Agricultural University, Urumqi, China

**Keywords:** Pear tree pruning, 3D structure analysis, Shoot characters, Automated pruning

## Abstract

The comprehensive understanding of the dormant pruning patterns in pear trees, along with the accurate identification of shoots suitable for pruning, is essential for implementing automated pruning and fruit production. Due to the complexity of tree architecture, previous descriptions of pruning strategies were qualitative summaries based on experience. In this study, we proposed a high-precision shoot extraction pipeline through point cloud alignment at different times, enabling a quantitative analysis of the pruning patterns. The structural parameters of 126 full bearing period pear trees, encompassing two cultivars and three architectures, were characterized, including the shoot number, single shoot angle and length, as well as shoot length density. The validation results demonstrated that the method attained an *R*^*2*^ of 0.82, 0.92, and 0.85 for shoot number, single shoot angle and length, respectively, with mean absolute error of 18.72, 6.08°, and 0.13 ​m. The findings indicate that tree architecture exerts a greater influence on pruning compared to cultivar, particularly in Cuiguan, where significant differences were observed across diverse tree architectures. The characters of the corresponding annual (one-year-old) shoots (AS) and pruned shoots (PS) exhibit similar distribution. The AS, constituted 78.62% of the PS number, and 94.90% of length of AS were pruned, indicating that dormant pruning in full bearing period pear tree primarily targets at the annual shoots, and the pruning of annual shoots is mainly by thinning. This study could help the automatic pruning system make pruning decisions and promotes the development of fine orchard management.

## 1 Introduction

The pear *(Pyrus)* is one of the most widely cultivated fruit trees in the world, with a history of cultivation spanning millennia and a range of cultivation extending across almost all temperate regions [1]. Pruning is an indispensable aspect of pear orchard management, as it plays a pivotal role in fostering fruit growth and canopy structure cultivation. Compared to summer pruning, dormant pruning is easier to operate, more obvious for fruit yield and quality improvement, and more beneficial for fruit tree canopy development [[2], [3], [4]]. Appropriate pruning can enhance the ventilation and light transmission conditions within the fruit tree canopy and facilitate a better balance between vegetative and reproductive growth. This can stimulate blossoming and fruiting while simultaneously maintaining and enhancing the yield and quality of the fruit. Furthermore, it can avert the phenomenon of alternate bearing, a common issue in pear trees characterized by significant yield fluctuations between consecutive years due to imbalances in vegetative and reproductive growth, and mitigate the risk of infestation by pests and pathogens. Nevertheless, the pruning of fruit trees has traditionally relied heavily on manual labor, particularly the need for skilled pruners, making it the most labor-intensive post-harvest activity in orchards, which has resulted in high yield costs, which accounts for more than 20% of the annual cost of crops such as apples [5] and pears [[6], [7]]. Moreover, as the labor force working in agriculture decreases each year, the costs are getting higher and higher, which has a significant negative impact on the development of orchards.

The advent of agricultural mechanization has brought pruning equipment into orchards, aiming to reduce manual labor or perform non-selective pruning. Especially, mechanized fruit tree pruning has been studied for many years. Early research in this field focused on bulk pruning, where a cutting bar was operated along orchard rows at a fixed distance from the center of the tree canopy, which restricted the canopy to a predetermined shape. However, this kind mass unselective pruning has been found to result in reduced fruit yield and quality [8].

In order to address the range of issues caused by non-selective pruning, researchers have conducted a series of studies in recent years with the aim of achieving intelligent selective pruning. The advancement of plant phenotyping techniques has led to a growing interest in the extraction of spatial structure information from branches, which is exactly the prerequisite for selective pruning. By combining RGB cameras with depth information to extract target branches, pruning decisions were developed by analyzing parameters such as branch length, inter-branch distance, and radius [9,[10], [11]]. However, due to the inherent limitations of camera performance, the reconstruction of branches and the extraction of parameters are only effective in areas with relatively simple branch structures, such as trellised architectures [12]. In more complex orchard environments and canopy regions with intricate branch structures, the accuracy of branch reconstruction and parameter extraction is significantly compromised due to limitations such as self-occlusion, which occurs when overlapping branches and leaves obstruct the camera's line of sight, resulting in incomplete or inaccurate imaging data [13].

The use of stereo cameras and LiDAR, as advanced 3D reconstruction technologies, can effectively address the limitations of RGB 2D imaging by accurately capturing and reconstructing the structural parameters of trees through combined multi-view perspectives [14,[15], [16]]. However, segmenting branches from three-dimensional point clouds remains significantly more challenging than performing segmentation in two-dimensional images due to the increased complexity and density of spatial data. Currently, two primary methods are employed for branch segmentation in fruit tree point clouds: (1) Skeletonization-Based Segmentation: After skeletonizing the point cloud, branches are segmented based on their hierarchical relationships and geometric attributes, such as branch angle, radius, and length. For fruit trees with relatively simple canopy structures, this method yields results comparable to those achieved from ground truth [[17], [18], [19]]. However, as canopy structures become more complex, the skeletonization results often appear disorganized, leading to reduced segmentation accuracy, which limits their application in supporting precise pruning decisions. (2) Deep Learning-Based Segmentation: Supervised deep learning networks have shown promising results in the segmentation of smaller herbaceous plants [[20], [21], [22], [23], [24]] and general tree branch and leaf structures [25,26]. However, research specifically focusing on branch segmentation in fruit trees using deep learning methods remains relatively limited. Due to the high similarity in the geometric features of branches across different hierarchical levels, current deep learning networks struggle to achieve accurate segmentation. Though in fruit trees with simpler canopy structures and fewer branches, the accuracy of branch segmentation can reach approximately 84% [27], which is sufficient for calculating structural parameters and supporting pruning strategies. But the accuracy decreases significantly in canopies with more complex structures. The increased number of branch layers, coupled with frequent overlapping and self-occlusion, introduces substantial challenges for deep learning-based segmentation methods, ultimately limiting their effectiveness in accurately identifying and segmenting branches.

Furthermore, current pruning decision-making methods are primarily based on specific structural parameters, including branch length, angles, and radius. However, these approaches often overlook the relationship between the pruning strategy and the distribution of annual (one-year-old) shoots, particularly in pear trees. Even in fruit tree physiology research, studies addressing this aspect remain limited [28]. However, understanding the growth dynamics of annual shoots is critical, as they directly influence both the structure of the canopy and the targets selected during pruning [29]. For instance, the physiological growth patterns of pear trees determine that annual shoots typically grow upright with large angles, aligning precisely with the characteristics of shoots suitable for pruning [30,31]. In recent years, there has been limited research on fruit tree pruning [29], while the application of 3D reconstruction tools, such as Terrestrial Laser Scanning (TLS), offers new possibilities for monitoring the growth dynamics of annual shoots in pear trees and advancing intelligent pruning technologies.

Overall, there are still numerous constraints associated with precision automated pruning of fruit trees. In complex orchard environments and canopies with intricate structures, obtaining accurate branch parameters and identifying optimal pruning targets remain significant challenges for achieving precise pruning. In this study, we proposed a registration-based branch segmenting method for pear trees by integrating the ideas of rigid registration [32,33] and non-rigid registration [34,35]. This method leverages the biological characteristic of full-bearing-period pear tree trunks—which, as perennial plants, exhibit minimal elongation or growth—to enable point cloud registration across different growth periods. By removing overlapping parts, it facilitates further extraction and segmentation of branches. The primary objective of this study is to summarize the manual pruning strategy with annual growth of shoots in pear trees with complex canopy structures using 3D point cloud analysis. Subsequently, the growth and pruning patterns will be derived. The specific objectives of this study are as follows:

(1) To propose a pipeline to automatically align pear tree point clouds before and after pruning to extract and measure characters of pruned shoots and annual shoots.
(2) To analyze the characteristics of the pruned shoots of pear trees across architectures and cultivars.
(3) To elucidate the fundamental principles of pruning operations, as well as to summarize the rules for manual pruning in full bearing period pear tree.

## 2 Materials and methods

**Fig. 1:**
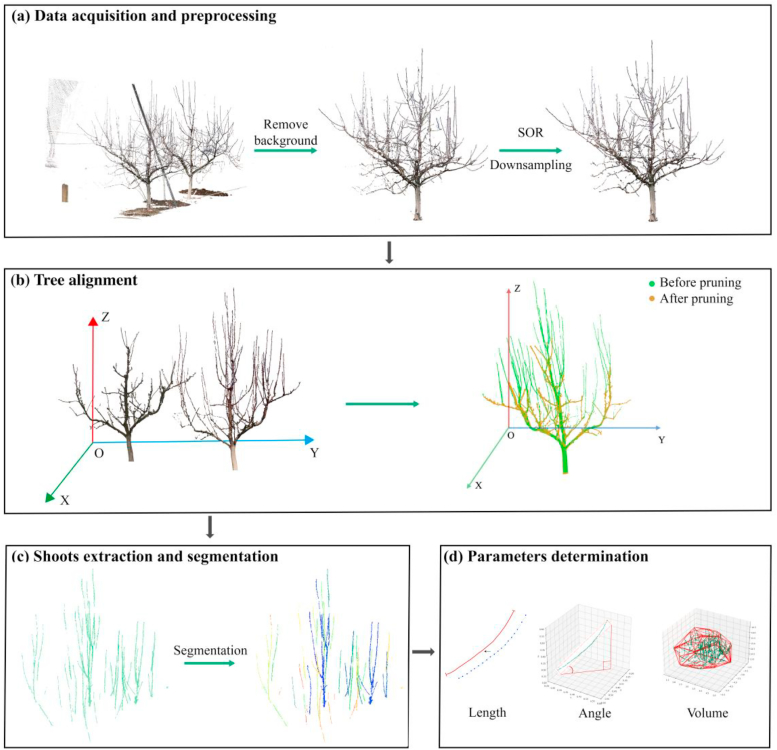
Research Workflow. Regions of Interest (ROIs) of single tree are manually selected from the raw point cloud data. The tree alignment module takes the same tree point clouds from different times as input and uses the tree with fewer points as a reference for alignment. The Shoots extraction and segmentation module processes the aligned trees to separate annual and pruned shoots to measure features.

The workflow (Fig. 1) of the pear tree shoot extraction and determination mainly includes four parts: point cloud data acquisition and preprocessing, pear tree trunk segmentation and alignment, shoot extraction and segmentation, and estimation of shoot parameters.

### 2.1 Data acquisition and preprocessing

#### 2.1.1 Pear tree point cloud data acquisition

**Fig. 2:**
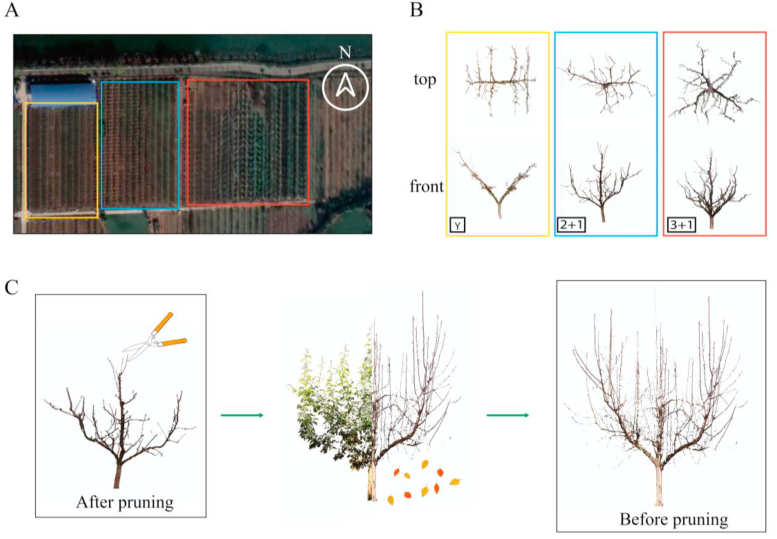
Experimental orchard and illustration of architecture and tree change with time. (A) and (B) show the three tree training systems, with colored blocks in (A) indicating planting positions corresponding to those in (B). (C) depicts the pear tree's structural state after pruning, during growth, and before the next pruning.

Two pear tree cultivars, 'Cuiguan' and 'Hosui', were studied, each exhibiting one of three training systems: Y, 2 ​+ ​1 and 3 ​+ ​1 architecture (Fig. 2A and B). These trees were planted in 2016 in the Jinling Pear Orchard, a commercial orchard in Nanjing, Jiangsu Province, China. High-precision point cloud data of the pear trees were acquired using a FARO Focus3D S70 three-dimensional laser scanner (FARO Technologies, Inc., Lake Mary, FL, USA).

The target trees were scanned several times during the study period (Fig. 2C). The first scan (BP_20_) was conducted in January 2020, before pruning. The second scan (AP_20_) took place in March 2020, immediately after pruning. The third scan (BP_21_) was performed in January 2021, before the subsequent pruning and after all leaves had fallen. This process was repeated in February 2023 (after pruning, AP_23_), November 2023 (before the subsequent pruning, BP_24_), and January 2024 (after pruning, AP_24_). Details of the dataset are presented in Table 1.

**Table 1:** Experimental data collection (2020–2024).

Data Name	Y		2 ​+ ​1		3 ​+ ​1		Total
	CG	HS	CG	HS	CG	HS	
BP_20_	2	2	3	6	3	4	20
AP_20_	2 (BP_20_)	2 (BP_20_)	3 (BP_20_)	6 (BP_20_)	3 (BP_20_)	4 (BP_20_)	20
	0 (BP_21_)	0 (BP_21_)	0 (BP_21_)	6 (BP_21_)	3 (BP_21_)	3 (BP_21_)	
BP_21_	N/A	N/A	N/A	6	3	3	12
AP_23_	9	11	9	9	N/A	N/A	38
BP_24_	9 (AP_23_)	11 (AP_23_)	9 (AP_23_)	9 (AP_23_)	0 (AP_23_)	0 (AP_23_)	56
	9 (AP_24_)	11 (AP_24_)	13 (AP_24_)	9 (AP_24_)	7 (AP_24_)	7 (AP_24_)	
AP_24_	9	11	13	9	7	7	56

**Fig. 3:**
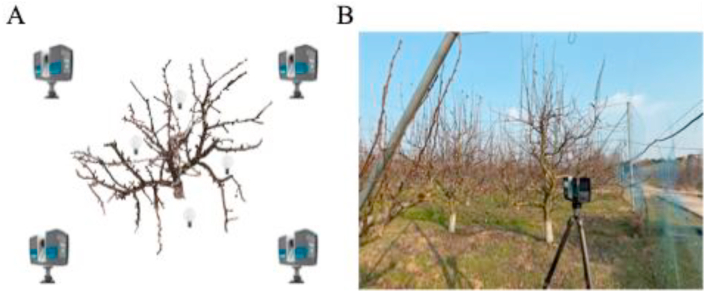
Field data acquisition setup. (A) Schematic diagram showing the layout of TLS (terrestrial laser scanning) stations and reflective targets. (B) Photograph of the field data collection setup.

All scanning sessions were conducted with either no wind or only a light breeze. To ensure comprehensive and accurate data collection, each tree was scanned from four different viewpoints surrounding the canopy to minimize occlusion and ensure structural completeness (Fig. 3A). The scanner was mounted on a tripod at an approximate height of 1.6 ​m (Fig. 3B). Multi-site point cloud registration was performed using FARO SCENE software (FARO Technologies, Inc., Lake Mary, FL, USA), which automatically aligns and merges the multi-view scans based on overlapping geometry and reference spheres placed in the scanning environment.

The table summarizes the number of paired trees, i.e., trees scanned at both the “After Pruning” (AP) and “Before Pruning” (BP) time points for each season. The scan pairing is indicated in parentheses — for example, in BP_23_, the entry ‘9 (AP_23_)’ indicates that 9 trees in data BP_24_ were also scanned in data AP_23_, allowing for paired analysis. CG representing “Cuiguan,” and HS representing “Hosui”. The “N/A” indicates that no scan was conducted at that time due to COVID-19 movement or orchard management restrictions.

#### 2.1.2 Data processing and validation data acquisition

Noisy point clouds and non-target objects are the primary factors affecting point cloud quality. To address this issue, CloudCompare (CloudCompare v2.12, [GPL software], [36]), an open-source 3D point cloud processing software, was utilized in this study. Non-target objects, such as other trees and bird netting in the orchard, were manually removed using the ‘Segment’ tool in CloudCompare. The lower part of each tree was uniformly cropped at a height of about 0.05 ​m to reduce noise from basal growth and ground clutter. In addition, suckers were either manually removed during field preparation or filtered out during point cloud preprocessing, ensuring minimal interference from undesired structures at the base of the trunk. Other noise elements, including ground-level vegetation and structural supports (e.g., Y architecture brace rods), were also manually removed during preprocessing to improve point cloud quality and alignment accuracy. Additionally, small clusters of noise points were eliminated using the Statistical Outlier Removal (SOR) algorithm with the neighborhood size set to 10.0 and the standard deviation multiplier set to 5.0, which effectively removes points that are significantly distant from their neighboring points. To optimize computational efficiency and reduce processing time, the point cloud data were down sampled using a voxel-based spatial subsampling method, with the voxel size set as 0.001 ​m.

For subsequent quantitative evaluations of phenotypic parameter estimations, point clouds of 24 pear trees were randomly selected (8 from each architecture) for manually measuring to establish ground truth values of shoot number and total shoot length. Additionally, to establish reference values for single shoot length and angle, 60 shoots were randomly selected for manual measurement. Specifically, two trees were randomly selected from each of the three tree architectures (Y, 2 ​+ ​1, and 3 ​+ ​1), and 10 shoots per tree were randomly sampled. All the manually measurements were conducted based on point clouds using CloudCompare. Single shoot length was determined by selecting a sequence of points along the shoot axis and summing the linear distances. For shoot angle, the orientation of each manually selected shoot was calculated using the principal axis of a fitted Oriented Bounding Box (OBB), following the same method used in the algorithmic pipeline, ensuring consistency in angle definition.

### 2.2 Segmentation of the trunk and point cloud alignment

**Fig. 4:**
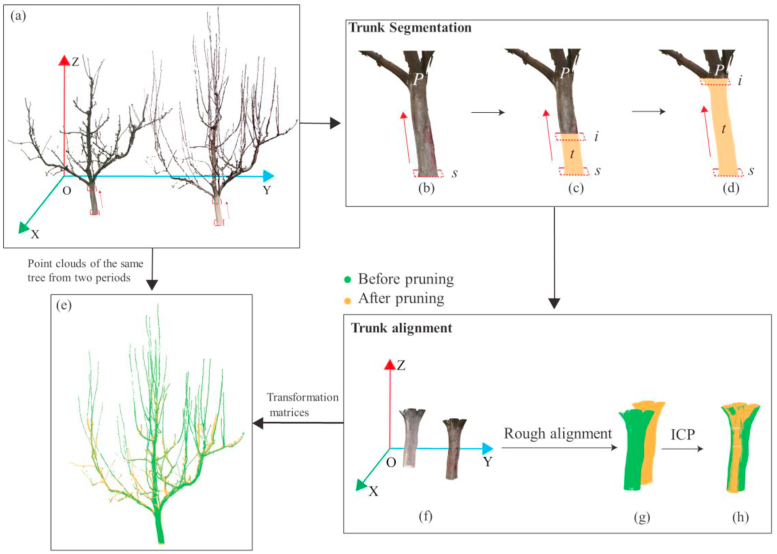
Trunk extraction and alignment**. (a)** The coordinates of point clouds scanned at different times show significant discrepancies; **(b)***S* represents the lowest slice of the point cloud; **(c)** Starting from the lowest slice, the algorithm gradually moves upward. The portions traversed during this process are classified as the trunk, with *i* representing the current slice being processed; **(d)** The movement stops once the specified threshold is reached; **(e)** Point clouds from different time points after registration; **(f)** Extracted trunk point clouds from different times; **(g)** Trunk point clouds after coarse registration; **(h)** Trunk point clouds after fine registration using the ICP algorithm.

Although the FARO terrestrial laser scanner can record GPS coordinates of target objects, the accuracy limitations of GPS positioning introduce spatial discrepancies between point cloud data of the same tree when scanned at different times (Fig. 4a). These positional errors affect the alignment and comparison of multi-temporal point clouds. Additionally, variations in canopy structure over time, caused by vegetative growth and pruning, further complicate direct point cloud registration across different times, making the process both challenging and computationally inefficient. The direct application of the Iterative Closest Points (ICP) algorithm often fails to achieve optimal registration results, as it tends to converge to a local optimum, preventing accurate alignment.

To address this issue, we proposed an algorithm to extract the entire trunks from the pear point clouds collected at different times. This algorithm employs a growing pipe model to simulate the trunk growth, starting at the base and progressively extending upward along the z-axis. When the pipe encounters a scaffold branch or a significant structural change that prevents smooth upward progression, it ceases to grow. The point cloud within the current pipe is then identified as the trunk. This segmentation approach ensures an accurate and consistent trunk extraction from complex tree point cloud structures (Fig. 4b, c, 4d).

The algorithm was defined by Algorithm.1. In this algorithm, *x* and *y* represent the widths in x and y coordinates at the base of the point cloud of trunk, defining its horizontal boundary. *λ* is a predefined threshold that ensure the trunk remains within reasonable bending limits.

Algorithm 1 Trunk extraction algorithm

**Table:** 

**Inputs:** Point cloud *P* **Parameters:** lowest layer *s,* current layer *i,* length *x,* width *y,* threshold λ **Outputs:** Point cloud of trunk *T* segmentation of pear tree point cloud into *n* layers *i* ​≤ ​*n, n← N*^*+*^ *T. push_back(s). i ← s* **while***x*_*i*_ ≤ *x*_*s*_*+ λ*, *y*_*i*_ ≤ *y*_*s*_*+ λ***do** *T. push_back(i)* Move upward along the direction of the trunk: *i = i+1* **end While**

After extracting the trunk of the pear trees (Fig. 4f), an initial coarse registration was performed by applying a translation transformation to align the overall coordinates of the point clouds from different times, as shown in Eq. (1). In this process, *A (x, y, z)* represents the average coordinates of the trunk point cloud after pruning, *B (x, y, z)* represents the average coordinates of the trunk point cloud before pruning, and *a (x, y, z)* represents the coordinate difference vector between the two trunk point clouds. This coarse registration step minimizes the global positional discrepancy between the point clouds, establishing a foundation for subsequent fine registration processes (Fig. 4g).

(1) A(x,y,z)=B(x,y,z)+a(x,y,z)

Next, the ICP algorithm was employed for fine alignment of the point clouds. This algorithm iteratively minimizes the Euclidean distance between corresponding points, progressively refining the spatial transformation relationship between the point clouds to achieve more precise alignment. After fine alignment, the point clouds of the same trunk from different times achieved maximum overlap, effectively reducing spatial discrepancies between temporal datasets (Fig. 4h). Finally, the transformation matrices obtained from both coarse registration and ICP alignment were applied to the entire pear tree point cloud, enabling the registration of pear tree point clouds across different times (Fig. 4e). This provides a reliable foundation for subsequent shoot extraction and pruning pattern analysis.

### 2.3 Shoots segmentation and recognition

**Fig. 5:**
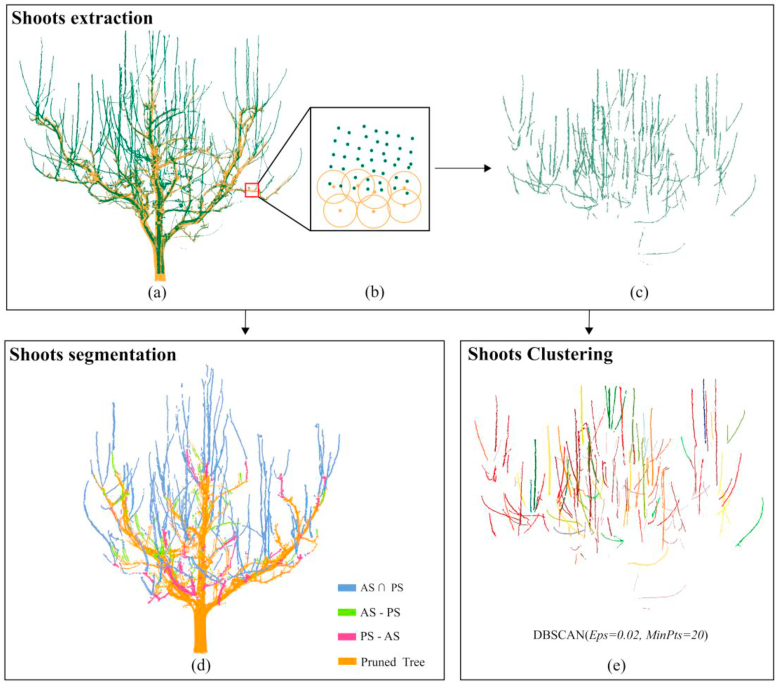
Shoots extraction and segment**ation. (a)** The yellow point cloud is designated as AP_20_, and the green point cloud is BP_20_; **(b)** The radius is 4 ​× ​d. Subsequently, the green point clouds that are less than the radius from the points in the yellow point cloud are removed, resulting in the **(c)** shoot point cloud; **(d)** “AS ∩ PS” refers to shoots that are both annual and pruned, “AS – PS” refers to the left part of annual shoots that were not completely removed, “PS – AS” refers to the part of branches that are pruned but not annual growing, and “Pruned Tree” refers to the tree point cloud after pruning.

By removing duplicate points from pear tree point clouds collected at different times, we aimed to extract the non-overlapping shoot regions (Fig. 5). Duplicate points were defined as points from two point clouds whose spatial distance falls below a predefined threshold, represented by the radius of yellow points (Fig. 5b). To quantify alignment accuracy and assess the degree of overlap, the Root Mean Square Error (RMSE) was calculated using all points from the pruned point cloud, each matched to its nearest neighbor in the un-pruned point cloud. The value of RMSE is denoted as ‘*d’*. Several thresholds were tested, ranging from 1 ​× ​*d* to 10 ​× ​*d*. A threshold of 4 ​× ​*d* was selected for shoot extraction, as it demonstrated the best separation performance. This threshold was used to identify and remove overlapping points between the before pruning and after pruning scans, thereby isolating the non-overlapping regions representing annual and pruned shoots with maximum completeness (Fig. 5c). Smaller thresholds failed to sufficiently remove portions of lateral or scaffold branches, leaving undesired overlaps. In contrast, larger thresholds compromised shoot continuity, leading to fragmentation and structural loss.

Annual shoots (AS) are identified as non-overlapping shoot regions between point clouds captured after pruning and before the next pruning (not in AP_i_ but in BP_i+1_, as shown in Fig. 2C), while pruned shoots (PS) are recognized as non-overlapping branch regions between point clouds taken before pruning and after the subsequent pruning (in BP_i+1_ but not in AP_i+1_). These are then input into the shoot extraction module to distinguish the overlapping and non-overlapping parts of branches: AS ∩ PS, the overlapping portion between annual shoots and pruning branches; AS - PS, where some parts of annual shoots were left after pruning; and PS - AS, referring to the part of branches that were not part of the annual shoot but were removed (Fig. 5d). The distribution of all type of shoots provides critical insights into annual growth variations and pruning patterns in pear trees. However, precise segmentation of these shoots is essential for further structural parameter analysis. To achieve this, the Density-Based Spatial Clustering of Applications with Noise (DBSCAN) algorithm was applied to the point cloud. DBSCAN, a density-based clustering algorithm, is widely used for point cloud segmentation due to its ability to identify clusters of arbitrary shapes and effectively handle noise [37]. Based on field observations and analysis of the point cloud data—and considering the down-sampling performed during preprocessing—we set the neighborhood radius parameter (Eps) to 0.02 ​m and the minimum number of points per cluster (MinPts) to 20 to achieve optimal clustering performance (Fig. 5e).

### 2.4 Measurements of shoots

#### 2.4.1 Shoot angle

**Fig. 6:**
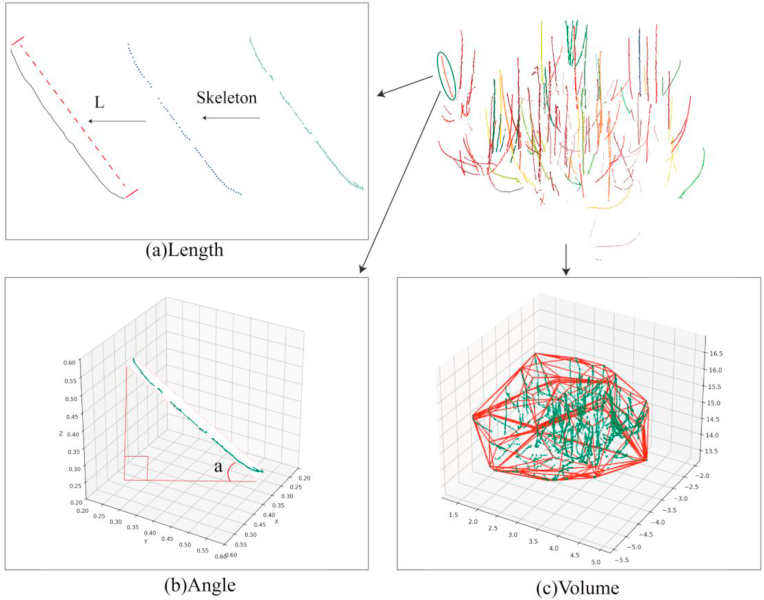
Determination of parameters from shoots point c**louds. (a)** The shoot length was calculated by summing the distances between neighboring points after skeletonization of the shoot point cloud. **(b)** The shoot angle was determined based on the inclination of the minimum enclosing box surrounding the shoot. **(c)** The volume of the canopy was calculated using the method of the minimum convex hull, which approximates the spatial boundary of the shoot.

In this study, the angle of a shoot is defined as the angle between the shoot and the horizontal plane (Fig. 6b). This definition was adopted to reflect the vertical inclination of shoots, which is a key trait influencing light capture, shoot vigor, and pruning decisions in orchard systems. While angles relative to the parent branch may offer more detailed structural insight, such measurements require precise topological information that is often unreliable in densely branched point clouds. In contrast, using the horizontal plane as a universal reference ensures consistent and interpretable angle measurements across shoots and trees [38]. Unlike perennial branches (e.g., scaffold and lateral branches), which have grown for longer periods and undergone treatments such as pulling, topping, and pruning to manipulate the direction of the branches and achieve the desired canopy structure, the majority of the pruned shoots and the annual shoots are relatively straight. Consequently, the angle of shoots can be accurately calculated using the representation of OBBs. By replacing relatively complex shoot point clouds with bounding boxes, computational efficiency and processing speed can be significantly improved. The angle of the shoot was calculated using the following formula:

(2) $$\theta =arccos(\frac{AB\cdot v}{∥AB∥\times ∥v∥})$$

Here *AB* represents the vector of the maximum edge of a shoot's bounding box, where *A* is the vertex at the base and *B* is the vertex at the top. The unit vector *v* lies on the *XoY* plane, aligned with the projection direction of vector *AB*.

#### 2.4.2 Shoot length

Although denoising and down-sampling were applied during preprocessing, the shoot point cloud data still contained an excessive number of points and exhibit clustering inaccuracies. These issues introduced errors in shoot length measurements when calculated based on the cumulative distances between adjacent points.

To address this problem, skeletonization was performed on the shoot point clouds using pc-skeletor [39], an open-source Python package specifically designed for point cloud skeletonization based on Laplacian contraction. This approach reduces data complexity while preserving the topological structure of the shoots, thereby enabling more accurate length measurements (Fig. 6a).

#### 2.4.3 Shoots length density

The length density of shoots is a key attribute of the inner canopy structure, and significantly influences a tree's capacity to intercept sunlight and plays a crucial role in its overall growth and productivity. To quantify this, the volume occupied by the canopy was calculated using the ‘compute_convex_hull’ function from the Open3D package [40]. In this context, the convex hull of a point cloud represents the smallest convex set that encompasses all points in the canopy point cloud. The shoot length density was defined as:

(3) D=L/V

Here *V(m*^*3*^*)* represents the convex hull volume of the canopy (Fig. 6c), and *L(m)* represents the total length of the shoots. This formula expresses shoot length density as the ratio of total shoot length to the convex hull volume, providing a quantitative measure of shoot distribution within the tree canopy.

#### 2.4.4 Quantitative metrics

The validation dataset constructed in Section 2.1.2 was used to evaluate accuracy. Linear regression analysis was performed to compare the estimated values extracted by the algorithm with the measured values gotten in 2.1.2 section. For shoot number and total shoot length, accuracy was assessed by comparing the total values between algorithmic and manual measurements based on the point clouds (see details in section 2.1.2). For single shoot length and angle, one-to-one comparisons were performed between manually identified shoots and their corresponding algorithm-segmented counterparts. Root Mean Square Error (RMSE) and Mean Absolute Error (MAE) were used to assess registration accuracy, while the coefficient of determination (R^2^) and RMSE served as metrics for parameter accuracy. Additionally, multiple comparisons were conducted to assess differences among cultivars and pear tree architectures. These metrics collectively provided a comprehensive evaluation of the accuracy of the proposed method.

## 3 Results

### 3.1 The accuracy assessment of alignment and segmentation

After successive coarse and ICP fine alignment of the two paired point clouds, the RMSE was calculated to assess the accuracy of the alignment [33], which was determined by calculating the distance between each point in one point cloud and its nearest corresponding point in the paired point cloud. The results indicate that the average RMSE across the entire tree decreased to 0.032 ​m following coarse and fine (ICP) registration.

**Fig. 7:**
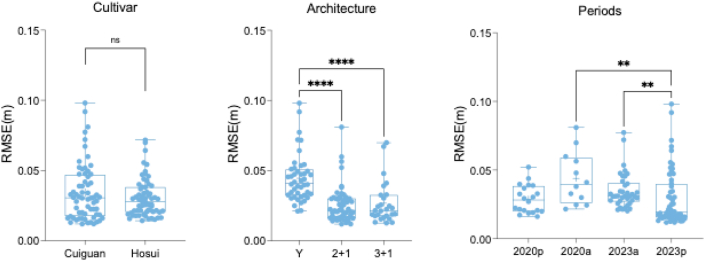
RMSE of point cloud alignment for different pear cultivars, different pear tree architectures and different time intervals. In Periods figure, “a” means the two paired trees which can extract the annual shoots; “p” means the two paired trees which can extract the pruned shoots. Different lowercase letters indicate significant differences, indicated by *p* ​≤ ​0.05. ‘ns’ indicates that the difference is not significant. The significance is indicated by ‘∗∗’ and ‘∗∗∗∗’ for *p* ​≤ ​0.01 and *p* ​≤ ​0.0001, respectively.

The registration error showed no significant difference between different cultivars, with average RMSE values of 0.034 ​m for Cuiguan and 0.030 ​m for Hosui (Fig. 7). However, there was a significant difference among different tree architectures (*p* ​< ​0.05), with the “2 ​+ ​1” architecture achieving the best performance, with a RMSE of 0.025 ​m. Another factor influencing registration accuracy was the time interval between the two paired point clouds. The registration accuracy of point clouds collected shortly before and after manual pruning was significantly higher compared to those collected over a year apart during natural growth. In 2020, the RMSE values for the point cloud data were 0.028 ​m and 0.044 ​m for 2020p and 2020a, respectively (*p ​< ​0.05*). Similarly, the RMSE values were 0.034 ​m and 0.029 ​m for the paired trees in 2023a and 2024p, respectively (*p ​< ​0.05*) (Fig. 7). Here, “a” refers to the pair of trees used to extract annual shoots, while “p” refers to the pair of trees used to extract pruned shoots. Specifically, 2020p refers to the pair of trees BP_20_ and AP_20_ (n ​= ​20), used to extract pruned shoots, while 2020a refers to the pair of trees AP_20_ and BP_21_ (n ​= ​12) used to extract annual shoots. Similarly, 2023a refers to the pair of trees AP_23_ and BP_24_ (n ​= ​38), used to extract annual shoots, and 2023p refers to the pair of trees BP_24_ and AP_24_ (n ​= ​56), used to extract pruned shoots. Due to the presence of significantly higher errors in some trees, which could result in inaccurate shoot segmentation, trees with a RMSE exceeding 0.09 ​m were excluded from further segmentation and analysis (n ​= ​2).

### 3.2 Estimation of shoot architecture parameters

**Fig. 8:**
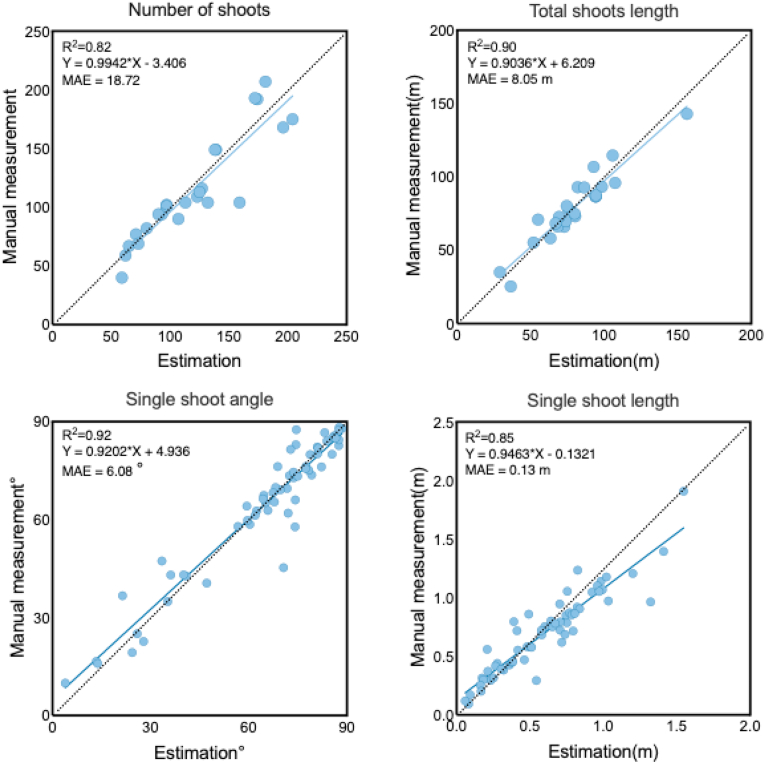
Shoots architectural trait estimation results and quantitative performance evaluation.

The parameter determination methods were evaluated based on shoot number, shoot length, and shoot angle. The estimated shoot number, total shoot length, and single shoot angle and length obtained using our method exhibited strong correlations with manual measurements, with R^2^ values of 0.82, 0.90, 0.92, and 0.85, respectively (Fig. 8). The corresponding MAE were 18.72, 8.05 ​m, 6.08°, and 0.13 ​m, respectively. These results indicate that the proposed method achieves high accuracy in both shoot counting and shoot angle and length estimation for pear trees.

Outliers were observed in the shoot number, particularly when the number of shoots was high. These outliers are likely attributable to segmentation errors caused by overlapping or closely spaced shoots. In such cases, DBSCAN may have either merged two adjacent shoots into one segment (under-segmentation) or split a single shoot into multiple fragments (over-segmentation). In contrast, the estimation of shoot length exhibited relatively higher accuracy. This is primarily because, despite potential inaccuracies in shoot segmentation, the length differences calculated by our algorithm remained minimal.

For single shoot angle estimation, outliers were primarily concentrated in regions with lower angles. This may be due to mutual occlusion among shoots with low angle, which can lead to segmentation inaccuracies and, consequently, larger errors in angle estimation. Additionally, shoots emerging from the lower canopy often exhibit shallow insertion angles and considerable curvature, both of which increase the difficulty of accurate angle measurement. The estimation accuracy for single shoot length was relatively low. Shorter shoots were slightly underestimated, mainly because threshold settings during segmentation led to partial loss of shoot length. In contrast, longer shoots showed relatively serious overestimation, which often occurred when the clustering algorithm mistakenly identified multiple separate shoots as a single one.

### 3.3 Characteristics of pruned shoots in different tree architecture and cultivar

**Fig. 9:**
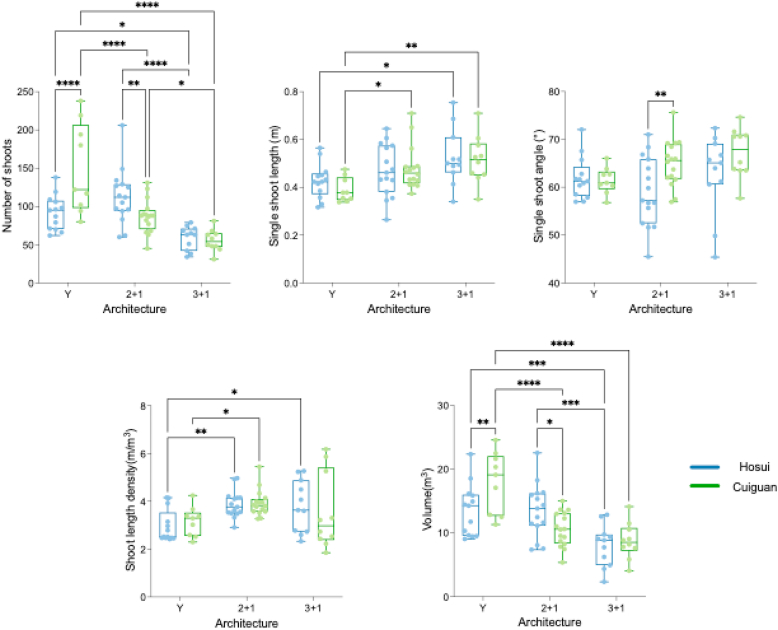
Pruning characteristics of pear trees across different tree architectures and cultivars. The pruned shoots data were from BP_20_ and AP_20_, as well as BP_24_ and AP_24_. The significance is indicated by ‘∗’, ‘∗∗’, ‘∗∗∗’*and* ‘∗∗∗∗’ *for P ​≤* ​0.05, P ​≤ ​0.01, P ​≤ ​0.001 and P ​≤ ​0.0001, respectively*.*

The pruned shoots analyzed in this section were obtained from BP_20_ and AP_20_ (n ​= ​20), as well as BP_24_ and AP_24_ (n ​= ​54). The methodology outlined in Section 2.3 was used to systematically segment the pruned shoots, followed by an analysis of pruning characteristics across cultivars (Cuiguan and Hosui) and tree architectures (3 ​+ ​1, 2 ​+ ​1, and Y). The results revealed significant differences in the number of shoots, individual shoot length, volume, and shoot length density across different tree architectures and cultivars (Fig. 9).

The number of pruned shoots varied significantly across tree architectures in Cuiguan, but not in Hosui. In Cuiguan cultivar, trees with the 3 ​+ ​1 architecture having the lowest shoot number (55.52), followed by 2 ​+ ​1 (87.13) and Y (146.70) architecture. However, in the Hosui cultivar, the difference in the number of pruned shoots between the Y and 2 ​+ ​1 architectures was not significant, although the 3 ​+ ​1 architecture still exhibited the lowest number of pruned shoots among three architectures (58.87).

Single shoot length and shoot length density were significantly influenced by tree architecture, with more noticeable effects in Cuiguan. However, no significant difference was found between the Hosui and Cuiguan pear trees. In Cuiguan, the Y architecture had the shortest shoot (0.385 ​m) compared to 3 ​+ ​1 (*p ​< ​0.01*) and 2 ​+ ​1 (*p ​< ​0.05*) architecture, while no significant difference was found between the 3 ​+ ​1 and 2 ​+ ​1 architectures. In Hosui, a significant difference was observed only between Y and 3 ​+ ​1 architectures (p ​< ​*0.05*). Similarly, in Cuiguan, Y architecture exhibited significantly lower shoot length density compared to 2 ​+ ​1 (*p ​< ​0.05*), while in Hosui, it was lower than both 2 ​+ ​1 (*p ​< ​0.01*) and 3 ​+ ​1 architectures (*p ​< ​0.05*), which may be influenced by the volume.

The volume of canopy also showed significant differences across tree architectures and cultivars, with Y architecture in Cuiguan exhibiting the largest volume (17.60 ​m^3^), while both cultivars in the 3 ​+ ​1 architecture exhibited the smallest volume.

Shoot angle differences across architectures and cultivars were minimal, with no significant patterns observed in this parameter.

### 3.4 Relationship between the pruned shoots with the annual shoots of the corresponding trees

**Fig. 10:**
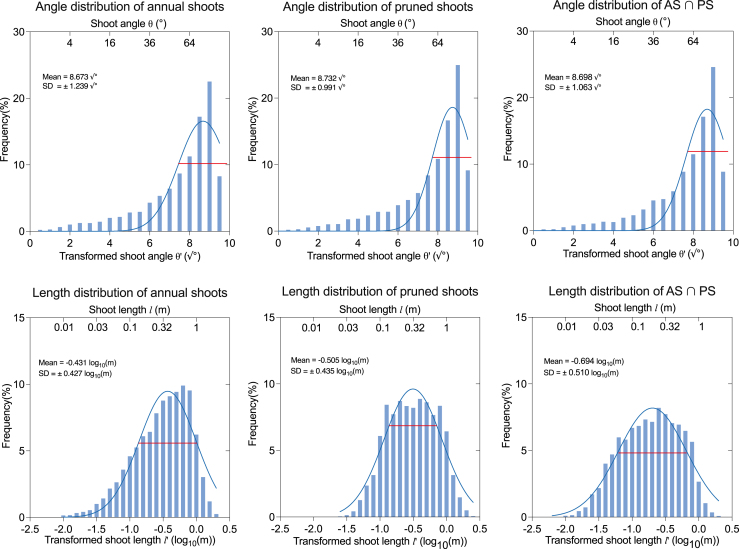
Illustrates the distribution of angles and lengths for both annual and pruned shoots. To perform normal fitting, the angle data were transformed using a square root transformation, while the length data underwent a logarithmic transformation. The red horizontal line in each plot represents the position corresponding to 1 standard deviation (SD) from the mean, providing a reference for the spread of the data.

From the spatial overlap analysis between annual shoots (based on AP_23_ and BP_24_, n ​= ​38) and pruned shoots (based on BP_24_ and AP_24_, n ​= ​38) of the same tree shown in Fig. 5d, it is evident that many pruned shoots correspond to the annual shoots, indicating that a large proportion of annual shoots were removed during pruning. Therefore, we analyzed the relationship between the pruned shoots with the annual shoots of the corresponding trees. The distributions of both angle and length deviate from normality in both pruned shoots and annual shoots. To address data non-normal distribution in original measurements, square root transformation (θ' ​= ​√θ) was applied to angle data, while length measurements underwent log10 transformation (*l*' ​= ​log10(l+0.1)) (Fig. 10). Normality of the transformed data distribution was assessed using Kolmogorov-Smirnov tests, with results indicating normality (p ​> ​0.05). Based on these results, Gaussian curve fitting was performed.

For the analysis of 4857 annual shoots, the results indicated that the mean of annual shoot angles were 75.22° ($\bar{\theta^{\prime }}$ ​± ​SD: 55.26°–98.25°), while the mean of lengths were 0.370 ​m ($\bar{l^{\prime }}$ ​± ​SD: 0.138–0.991 ​m). An analysis of 5208 pruned shoots revealed that the mean value of the pruned shoot angle was 76.25° ($\bar{\theta^{\prime }}$: ±SD: 59.93°–94.54°), while the mean value of the pruned shoot length was 0.313 ​m ($\bar{l^{\prime }}$ ​± ​SD: 0.115–0.851m). A comparative analysis between the two datasets revealed that pruned shoots tend to have large angles (more than 71.93° in 60% shoots), with small angled shoots being less likely to be pruned. The distribution of shoot angle of the annual and pruned shoots is similar, and the distribution of shoot length of pruned shoots looks closer to normalized distribution than the annual shoots. These findings suggest that the pruning pattern largely depends on the growth of the annual shoots.

Additionally, further analysis of the AS ∩ PS revealed that the average angle was 75.65° ($\bar{\theta^{\prime }}$ ​± ​SD: 58.29°–95.28°), while the average length was 0.202 ​m ($\bar{l^{\prime }}$ ​± ​SD: 0.063–0.654 ​m). The angles of AS ∩ PS were more concentrated at larger values, suggesting that the annual shoots were more likely to be pruned at larger inclination angles. In terms of shoot length, although the distribution of the pruned shoots with annual shoots was similar, longer shoots (shoot length > 1m) accounted for a smaller proportion of the total annual shoots of the AS ∩ PS. It appears to be due to a shift in pruning strategy: for some very long shoots, the pruning method changed from thinning to heading back, resulting in the decrease of a proportion of longer shoots.

### 3.5 The pruning pattern of manual pruning operations

**Fig. 11:**
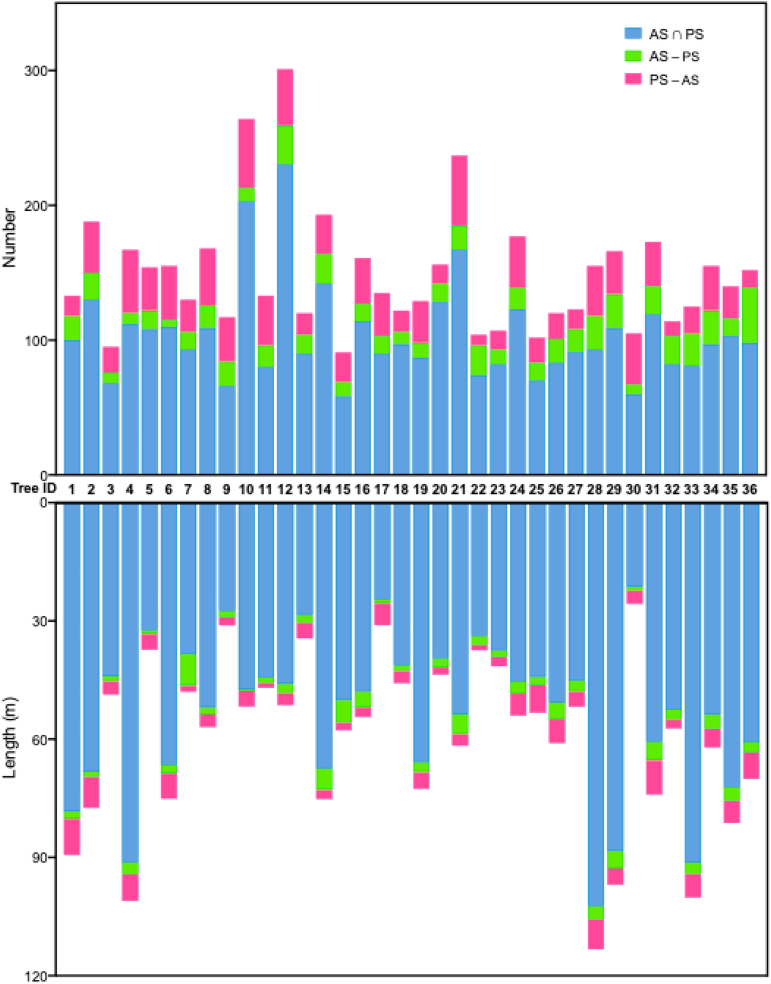
Total length and number of pruned shoots in relation to the length and number of annual shoots. Where trees with ID 1∼18 are Y architecture and with ID 19∼36 are 2 ​+ ​1 architecture. The data for the annual shoots is from AP_23_ and BP_24_, while the data for the pruned shoots is from BP_24_ and AP_24_.

By analyzing the pruned shoots with the annual shoots of the corresponding trees in dataset AP_23_, BP_24_ and AP_24_, we found that on average, 85.79% (by number) and 94.90% (by length) of annual shoots were pruned, with only a small fraction retained for future structural training (Fig. 11). These suggested that pruning in the experimental orchard followed a consistent pattern, primarily targeting annual shoots for tree shaping, with thinning as the main method for pruning annual shoots.

An analysis of the total lengths and angles of both pruned and annual shoots revealed a striking similarity in their structural parameters. In the experimental orchard, pruning practices-specifically the distributions of shoot length and angle-remained highly consistent and uniform over the two years of observation (2020 and 2023, Fig. S3 and Fig. 10). The AS ∩ PS (annual and also pruned), AS-PS (annual but not pruned), PS-AS (pruned but not annual) were separated as three classes. Further analysis of the relationship between annual and pruned shoots showed that, across consecutive growing seasons and pruning cycles (based on AP_23_, BP_24_, and AP_24_), an average of 78.62% of the pruned shoots were annual shoots, comprising 92.20% of the total pruning length (Fig. 11). Data from BP_20_, AP_20_, and BP_21_ were omitted in assessing the influence of annual shoot growth patterns on pruned shoot distribution, due to the data sequence (extracting pruned shoots first, then annual shoots). In addition to annual shoots, some perennial shoots were also removed during pruning. Although their proportion in the overall pruning process was relatively low (21.38% by count and 7.80% by length), they nonetheless played a crucial role in shaping the structural of pear trees.

## 4 Discussion

Dormant pruning of fruit trees is not an isolated operation in orchard management but must be considered within the broader context of the tree growth dynamics. Unlike summer pruning, dormant pruning requires precise segmentation of branches and parameter acquisition are essential preparatory steps [41,42]. The proposed alignment method synthesizes the concepts of segments-based registration alignment and the ICP algorithm, effectively achieving precise alignment of pear tree point clouds across different times. However, the accuracy of point cloud registration is influenced by branch displacement and deformation over time, which are primarily affected by both the time interval between data acquisitions and the quality of the 3D point clouds (Fig. S2). Then, by removing overlapping parts in paired point clouds, the shoots can be extracted by DBSCAN. Due to occlusion between shoots, segmentation errors occasionally occurred. Nevertheless, the precision of shoot segmentation was sufficient (see the example in Fig. S1 and Table S1) to support the phenotypic analyses presented in this study. The proposed method represents a significant advancement in the field of annual shoot and pruned shoots extraction and segmentation, particularly in the structurally complex full-bearing pear trees, compared with the traditional algorithms [15,17] and deep learning methods [14,22,27] which applied to relatively simple structured trees. However, our method also exhibits a decline in parameter measurement accuracy as the complexity of the canopy structure increases. As shown in Fig. 8, in shoot number detection, accuracy decreases as the number of shoots increases. This phenomenon primarily observed in some trees of Y architecture, with lower registration accuracy (Fig. 7) and higher shoot number (Fig. 9). This reduction in accuracy may be attributed to noise interference from tree supports and other obstructions within the Y architecture point clouds, leading to a decline in point cloud quality.

Besides the number, angle and length of the branches, diameter is also one meaningful structural trait, particularly in relation to making pruning strategy [43]. However, in the current study, branch diameter was not included in the analysis primarily due to the difficulty in defining a representative diameter value for irregular and tapering branches. In 3D point clouds of field-grown trees, the cross-sectional radius can vary considerably along the branch, and the presence of occlusion further complicates accurate diameter estimation. Incorporating robust, multi-scale diameter estimation methods is part of our future research plan, particularly in linking branch diameter with annual shoot development in different architectures.

Through a comparative analysis of pruned shoots across different cultivars and tree architectures, we observed significant differences in pruning patterns among tree architectures, but no significant differences between the cultivars. Regarding shoot number, Cuiguan exhibited a trend where the number of pruned shoots decreased as the number of scaffold branches in the tree architecture increased, a pattern not as pronounced in Hosui. In terms of the pruned single shoot length and shoot length density, no significant differences were found between cultivars, with tree architecture being the only factor influencing these traits. As the number of scaffold branches increased, the pruned single shoot length and shoot length density exhibited a tendency to increase for Y, 2 ​+ ​1, and 3 ​+ ​1 architecture, which had 2, 3, and 4 scaffold branches, respectively. This trend in shoot length density changes may be influenced by the volume of the tree architecture, as significant volume differences exist between architectures. These results suggest that tree architecture exerts a greater influence on shoot traits and pruning patterns than cultivar. This may occur because tree architecture governs the spatial distribution of branches, light interception, and shoot growth direction—all critical factors in apical dominance and lateral bud activation [38]. While cultivar-specific differences such as internode length and shoot vigor certainly exist, these factors may be overridden by the architectural constraints and training systems applied, particularly in mature, full-bearing trees.

Prior to this study, there had been a paucity of research conducted on the annual growth variation of fruit tree shoots [28]. By comparing the annual variation of shoots to pruned shoots, our study indicates that in pear trees which in full bearing period, the primary targets of dormant pruning were the annual shoots that grew in the previous year. The annual shoots constituted 78.62% of the pruned shoot number and 92.20% of the pruned length, and the majority of these shoots exhibited large angles (more than 71.93° in 60% shoots) and small angled shoots being less likely to be pruned. In addition, 85.79% (by number) and 94.90% (by length) of annual shoots were pruned. This finding suggests that the primary pruning targets for full-bearing pear trees are upright-growing annual shoots, and thinning is the main method for pruning annual shoots. By focusing on identifying and pruning annual shoots, most of pruning tasks can be effectively accomplished. This insight could be valuable for automated pruning research, by simplifying the development of automated pruning strategies. In addition, this approach could also be applied to other deciduous fruit trees, such as apples and peaches, to reveal the pruning strategy during dormancy. This could greatly accelerate research on automated pruning systems.

However, this study only focuses on monitoring of annual variation in shoots for the dormant pruning. While the pruning of annual shoots constitutes the majority of fruit tree pruning, the role of other branches should not be overlooked. Therefore, the division of perennial branches of fruit trees represents a subsequent step in this process. Moreover, the analyzed pear trees are in full bearing period. The growth and pruning rules will be further verified and investigated in before fruiting and early fruiting pear trees. In addition, variability in pruning practice between workers and differences in environmental conditions at the time of pruning may have introduced subjective biases. These potential sources of variation require further controlled observation to validate the generality of the observed pruning strategies.

## 5 Conclusions

We proposed a method that achieves high-precision extraction and characterization of pruned shoots and annual shoots in pear trees through accurate point cloud registration. The method enables a detailed evaluation of the pruning and growth characteristics of pear shoots. Moreover, this approach could be extended to other deciduous fruit trees with similar pruning system.

Tree architecture had a greater impact than cultivar among the three tree architectures and two pear cultivars studied. Particularly in Cuiguan, significant differences were found in the number of pruned shoots, single shoot length, canopy volume, and shoot length density across different architectures.

The characters between the annual shoots and pruned shoots reveal that, dormant pruning in full bearing period pear trees primarily targets shoots that emerged during the previous growing season, accounting for 78.62% by number and 92.20% by length of pruned shoots, with more than 71.93° of shoot angle in 60% pruned shoots. The similar distribution of characters of the annual and pruned shoots suggests that the pruning pattern largely depends on the growth of shoots during the previous growing season. In addition, on average, 94.90% of length of annual shoots were pruned, suggesting that the pruning of annual shoots is mainly by thinning.

However, due to differences in pruning systems and tree age, the dormant pruning strategy may need to be adjusted. This study could help the automatic pruning system make pruning decisions and promotes the development of fine orchard management.

## Author contributions

J.L., Y.M., and S.N. conceived the study; J.L. developed and tested the method, conducted experiments, analyzed the results, and wrote the original draft. J.L., G.W., H.X., and H.S. contributed to point cloud data acquisition. S.T., K.Q., H.Y., and S.Z. provided materials and technical support for the experiments. Y.M., W.G., and S.N. conducted the supervision and performed revisions of the manuscript. All authors read and approved the final manuscript.

## Funding

This work was co-financed by the Major Science and Technology Projects of Xinjiang Uygur Autonomous Region (2024A02006-3), the Jiangsu Agricultural Science and Technology Innovation Fund (No. CX (22)2025 and No. CX (23)1011), and the National Natural Science Foundation of China (No. 32001980).

## Data availability

The source code and point clouds samples used in this study are publicly available at: https://github.com/Lixiao-bai/Pear_branch_seg_and_analysis. Additional data can be made available upon reasonable request.

## Declaration of competing interest

The authors declare the following financial interests/personal relationships which may be considered as potential competing interests:Given their respective roles as Editor-in-Chief and Senior Editor, Seishi Ninomiya and Wei Guo had no involvement in the peer review of this article and had no access to information regarding its peer review. Full responsibility for the editorial process for this article was delegated to another journal editor.
