# Supplementary material for: Structural parameter determination and pruning pattern analysis of pear tree shoots for dormant pruning
Source: Plant Phenomics. 2025 Nov 13;7(4):100136. doi: 10.1016/j.plaphe.2025.100136 (PMC13109299; doi:10.1016/j.plaphe.2025.100136)
Supplement: Multimedia component 1 [file mmc1.docx]

**1 Data sample for branch segmentation**

To further clarify our segmentation process, we have included an example figure (Figure S1) showing the clustering result and provided a link to a point cloud sample used in the experiment in txt format (<https://github.com/Lixiao-bai/Pear_branch_seg_and_analysis>).


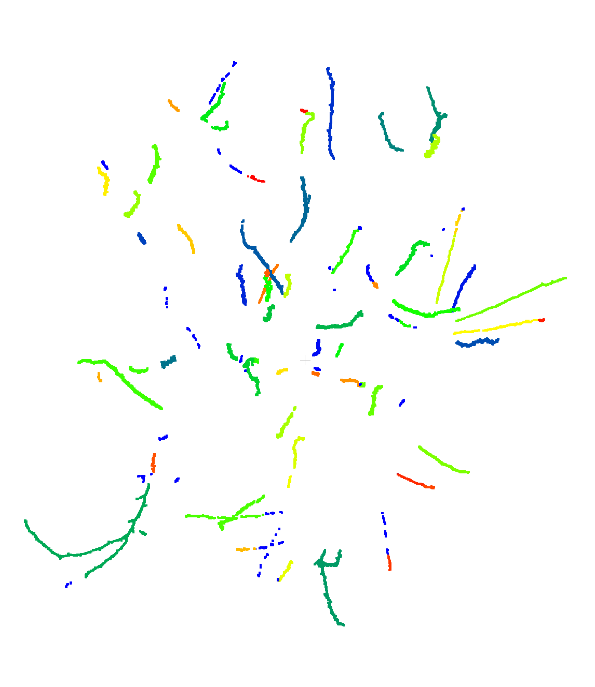


Fig. S1 Top View of the Segmented Point Cloud

**2 Evaluation of branch segmentation**

In order to better illustrate the occurrence of segmentation errors, we selected a pear tree with largest error and another one with average error in shoot counting, and calculated the point-level mean coverage (mCov), and instance-level precision and recall, respectively. It indicates that the counting error was largely dependent on the single-branch segmentation error. Even for the worst segmentation case, the precision is 0.779, which means it could be used for length and angle determination of single branch.

Table S1 Evaluation of single branch segmentation on two trees

| Tree ID | mCov | Precision | Recall |
| --- | --- | --- | --- |
| W20-1 (worst) | 0.503 | 0.779 | 0.499 |
| W17-13 (average) | 0.805 | 0.941 | 0.814 |

For the evaluation of single leaf segmentation, the mean coverage (*mCov*) was used. *mCov* represents the average point-level *IoU* matching between predicted and ground truth instance, which is defined as follows:

$$IoU=\frac{TP}{TP+FP}$$

$$mCov(I, P)=\frac{1}{I}\sum_{m=1}^{1} \max_{n} (IoU(I_{m}{,P}_{n}))$$

Where *I* represent the number of all instances, $I_{m}$ represents the real point set of the *m*th instance, and $P_{n}$ represents the predicted point set of the *n*th instance. The calculation of *IoU* is the same as that in semantic segmentation.

In addition to point-level evaluation, all instances with *IoU* higher than 0.5 were counted and evaluated at the instance level using two metrics: *Precision* and *Recall*. The definitions are as follows:

$$Precision=\frac{T}{P}$$

$$Recall=\frac{T}{G}$$

where *T* is the number of *IoUs* greater than 0.5 in predicted instances and manually marked instances, *P* is the total number of predicted instances, and *G* is the number of manually marked instances.

**3 Error Visualization of the Registered Point Clouds**

While we used rigid alignment, local non-rigid deformation could indeed contribute to residual errors (Fig. S2). The accuracy of point cloud registration is influenced by branch displacement and deformation over time, which are primarily affected by both the time interval between data acquisitions and the quality of the 3D point clouds (Fig. S2).


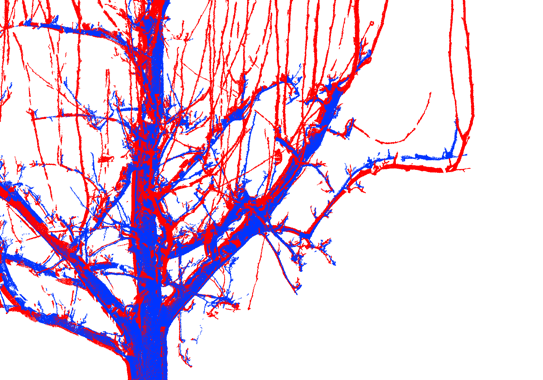


Fig. S2 Error visualization of the registered point clouds.

**3 Error Visualization of the Registered Point Clouds**

Although the data from BP_20_, AP_20_, and BP_21_ could not be used in assessing the influence of annual shoot growth patterns on pruned shoot distribution, the angle and length distribution of the pruned shoots were analyzed (Fig. S3).

Fig. S3 The distribution of angles and lengths for pruned shoots (data from 2020).
